# Observations on significant hemodynamic changes caused by a high concentration of epidurally administered ropivacaine: correlation and prediction study of stroke volume variation and central venous pressure in thoracic epidural anesthesia

**DOI:** 10.1186/s12871-017-0444-x

**Published:** 2017-11-16

**Authors:** Jeong-Min Hong, Hyeon Jeong Lee, Young-Jae Oh, Ah Rhem Cho, Hyae Jin Kim, Do-Won Lee, Wang-Seok Do, Jae-Young Kwon, Haekyu Kim

**Affiliations:** 1 Department of Anesthesia and Pain Medicine, Pusan National University Hospital, 179 Gudeok-Ro, Seo-Gu, Busan, 602739 South Korea; 2 Medical Research Institute, Pusan National University Hospital, Busan, South Korea

**Keywords:** Epidural, local, ropivacaine, Epidural administration, Stroke volume variability

## Abstract

**Background:**

Thoracic epidural anesthesia (TEA) exacerbates hypotension due to peripheral vasodilator effects following the use of general anesthetics. This study aimed to compare the hemodynamic changes caused by three different concentrations of epidural ropivacaine and to evaluate the performance of the stroke-volume variation (SVV) and central venous pressure (CVP) during TEA with general anesthesia.

**Methods:**

A total of 120 patients were administered 8 mL of ropivacaine solution via epidural injection, following randomization into one of three groups based on the concentration of ropivacaine in the study solution: 0.75%, 0.375%, or 0.2%. Hemodynamics were monitored for 30 min after loading. We analyzed the hemodynamic changes in the subgroups according to an age cutoff of 60 years. Receiver operating characteristic (ROC) analysis was performed to characterize the relationship of the SVV, CVP, and a 20% decrease in the mean arterial pressure (MAP) following TEA.

**Results:**

Data from 109 patients were analyzed. MAP and systemic vascular resistance index were significantly decreased, and SVV was significantly increased after epidural loading only in the 0.75% ropivacaine group. There was a significant difference in hemodynamics between young and elderly subgroups in the 0.75% ropivacaine group. SVV showed a negative correlation with MAP, whereas CVP showed no correlation. The ROC analysis of SVV demonstrated a weak predictive ability of a 20% decrease in MAP at 10 min after the loading dose, with an area-under-the-curve of 0.687 and a 9.5% optimal cutoff value (sensitivity, 60.6%; specificity, 68.9%).

**Conclusions:**

A high concentration of ropivacaine through TEA caused a significant decrease in the systemic vascular resistance and blood pressure. More significant decreases were shown in the elderly patients. Though the change of SVV showed a negative correlation with hypotension and indicated functional hypovolemia after TEA, the predictability was limited.

**Clinical trials registration:**

Number: NCT01559285, date: January 24, 2013.

## Background

Combining thoracic epidural analgesia (TEA) with general anesthesia has been widely used for perioperative treatment in patients undergoing major upper abdominal surgery. TEA with general anesthesia provides pain relief, reduces metabolic and hormonal stress, and promotes recovery of gastrointestinal function, thereby reducing recovery time after surgery [1–4].

However, TEA under general anesthesia leads to more hemodynamic impairment compared to general anesthesia alone. Hemodynamic impairments accompanying TEA with general anesthesia are affected by various factors. The extent of sympathetic denervation, balance of sympathetic and parasympathetic activity, and pharmacological effect of systemically absorbed local anesthetics (LA) are considered to be contributing factors [5].

Although the most common physiologic consequence of epidural anesthesia is hypotension, primarily due to the sympathetic nervous system block [6], clinical studies on hemodynamic effects of epidural local analgesic-related dose titration leading to different cardiovascular responses have not been well-studied.

In mechanically ventilated patients, arterial pressure waveform analyses can characterize the interaction between the peripheral arterial blood flow and the respiratory cycle by employing variations in systolic pressure, pulse pressure, and stroke volume [7]. Stroke-volume variation (SVV) has been shown to be an accurate index of fluid responsiveness [8].

Most cardiac output devices require calibration to adjust for the patient’s vascular tone change. Thus, their use is limited owing to abrupt changes of vascular tone during epidural or spinal anesthesia. In contrast, the FloTrac™/Vigileo™ system (Edwards Lifesciences, Irvine, CA) does not need any calibration because it continuously adjusts for the patient’s vascular tone change by using a novel algorithm incorporated within the Vigileo monitor, which is applied to the digitized arterial pressure wave [9]. Currently, only a few clinical studies have demonstrated the predictability of SVV on hemodynamic changes following TEA combined with general anesthesia.

In the current study, we tested whether hypotension following TEA is more significant when using a higher concentration of LA, which was administered to patients undergoing major upper abdominal surgery. In addition, we evaluated whether SVV could be a diagnostic parameter for the detection of hypotension after TEA combined with general anesthesia.

## Methods

### Patients

This study was registered with Clinicaltrials.gov (NCT01559285). After approval by the IRB of the Yangsan Hospital of Pusan National University, this prospective, randomized, and double-blind study was carried out in 120 patients, 18 to 65 years of age, undergoing major upper abdominal surgery using combined TEA and general anesthesia after obtaining informed consent. Medical histories and physical examinations were obtained for all subjects before admission into the study. Exclusion criteria included: known significant cardiac or respiratory disease, cardiac arrhythmia, neurological dysfunction, or a contraindication for regional anesthesia. The patients were randomized to receive one of three different concentrations of the study solution in 8 mL: 0.75% ropivacaine (60 mg), 0.375% ropivacaine (30 mg), or 0.2% ropivacaine (16 mg). Random numbers were generated by a computer and used to allocate the subjects into three groups. The allotment took place after induction of anesthesia. The study solution was prepared and blinded by an anesthetic nurse investigator, and therefore, the induction anesthesiologist was unaware of the drug concentration.

### TEA and general anesthesia

After fasting for more than 8 h, all patients were premedicated with intramuscular glycopyrrolate (0.2 mg). On arrival in the operating room, lactated Ringer’s solution (10 mL/kg) was administered to compensate for the overnight fast, urinary losses, and evaporative losses.

A 20-gauge epidural catheter was inserted towards the cephalad direction at the T10–T11 space via an 18-gauge Touhy needle in the lateral decubitus position. The epidural space was confirmed by the loss of resistance technique. After negative aspiration for cerebrospinal fluid or blood, a 3 mL test dose of 2% lidocaine with 15 μg epinephrine was injected to detect intravenous misplacement or unintended subarachnoid catheter placement.

General anesthesia was induced with 2 mg/kg of propofol. An endotracheal tube was inserted after injection of 0.8 mg/kg of rocuronium. Routine perioperative monitoring was conducted to acquire the heart rate (HR), pulsed oxygen saturation, electrocardiograph, mean arterial blood pressure (MAP), end-tidal carbon dioxide, end-tidal anesthetic concentration, and bispectral index scale (BIS). After induction, the radial artery cannulation with a 20-gauge cannula (BD Angiocath Plus, Becton Dickinson, Singapore) was connected to a FloTrac™/Vigileo™ system. Thereafter, central venous pressure (CVP) was monitored through a central venous catheter (BD Careflow™ 7Fr 150 mm, Becton Dickinson, Singapore), which was placed into the right subclavian vein.

Maintenance of anesthesia was conducted with 1.0–1.5 MAC vol% of sevoflurane to achieve BIS values between 40 and 60. The lungs were mechanically ventilated with tidal volumes of 8 mL/kg at 10–12 breaths per minute using an air-oxygen mixture with a fraction of inspired oxygen concentration of 50% and a fresh gas flow of 2 L/min.

### Monitoring and data collection

SVV, variation of beat to beat stroke volume, was measured by an arterial line using the FloTrac™/Vigileo™ system using this equation during the most recent 20 s: SVV (%) = (SVmax − SVmin)/SVmean.

Cardiac index (CI), stroke volume index (SVI), and systemic vascular resistance index (SVRI) were also recorded and calculated using the FloTrac™/Vigileo™ system. All hemodynamic measurements were recorded at baseline (T0), 10 min (T10), 20 min (T20), and 30 min (T30) following administration of an epidural drug before surgical incision. Hypotension (MAP <60 mmHg) and bradycardia (HR <50 bpm) were treated by a 10 mg ephedrine injection.

### Subgroup analysis of hemodynamic changes

For each group, the patients were divided into two subgroups by a cutoff age of 60 years to perform the age-based analysis. The acquired hemodynamic data after administration of the epidural loading doses were compared and analyzed between the young and elderly subgroups in each group.

### Statistical analyses

The primary endpoint was the change in the MAP after administration of the epidural loading dose. The power analyses showed that 36 patients would be necessary for a beta = 0.2 and alpha = 0.05. For compensation of 10% of the potential dropouts, we enrolled 40 patients in each group. The sample size was estimated from the preliminary data acquired from 10 patients, and an assumption that 10 mmHg differences among the groups in the MAP would be clinically relevant after the onset of an epidurally administered ropivacaine effect.

Parametric data such as age, weight, height, fasting time, and infused volume of crystalloid were analyzed by the analysis of variance (ANOVA). The chi-square test was used to compare the American Society of Anesthesiologists (ASA) physical status, sex, type of surgery, and number of patients needing ephedrine. Body surface area (BSA) was calculated using the du Bois formula (BSA = body weight [kg]^0.425^ × body length [cm]^0.725^ × 0.007184). MAP, HR, CVP, SVV, SVI, CI, and SVRI were analyzed by two-way repeated measures ANOVA for group comparison after the sphericity test. BIS scales and end-tidal sevoflurane concentration were also analyzed by two-way repeated measures ANOVA. For group comparison at the measured time points (T0, T10, T20, and T30), the ANOVA with Bonferroni’s test was performed as a post hoc test. Throughout the observation period, the serial change of hemodynamic data was compared by ANOVA with Bonferroni’s test. Analysis to compare hemodynamic data measured in two subgroups with young and elderly patients in each group were also performed.

A correlation analysis was used for evaluation of the interaction between MAP and other hemodynamic variables in each group.

### Receiver operating characteristic analysis

All patients were divided into two groups based on the percent decreases in MAP following an epidural loading dose, with responders who showed more than a 20% decrease in MAP at T20, the lowest time point of MAP after epidural loading, and non-responders who showed less than a 20% decrease in MAP. Receiver operating characteristic (ROC) curves were obtained for SVV by varying the discriminating threshold of the variable and areas under curves (AUC) of ROC were acquired. A value of *P* < 0.05 was defined as statistically significant and data are presented as mean ± standard deviation (SD). All analyses were carried out using StatView version 5.0 (SAS, Chicago, IL, USA) and MedCalc® version 9.3.1 (MedCalc Software, Mariakerke, Belgium).

## Results

**Fig. 1:**
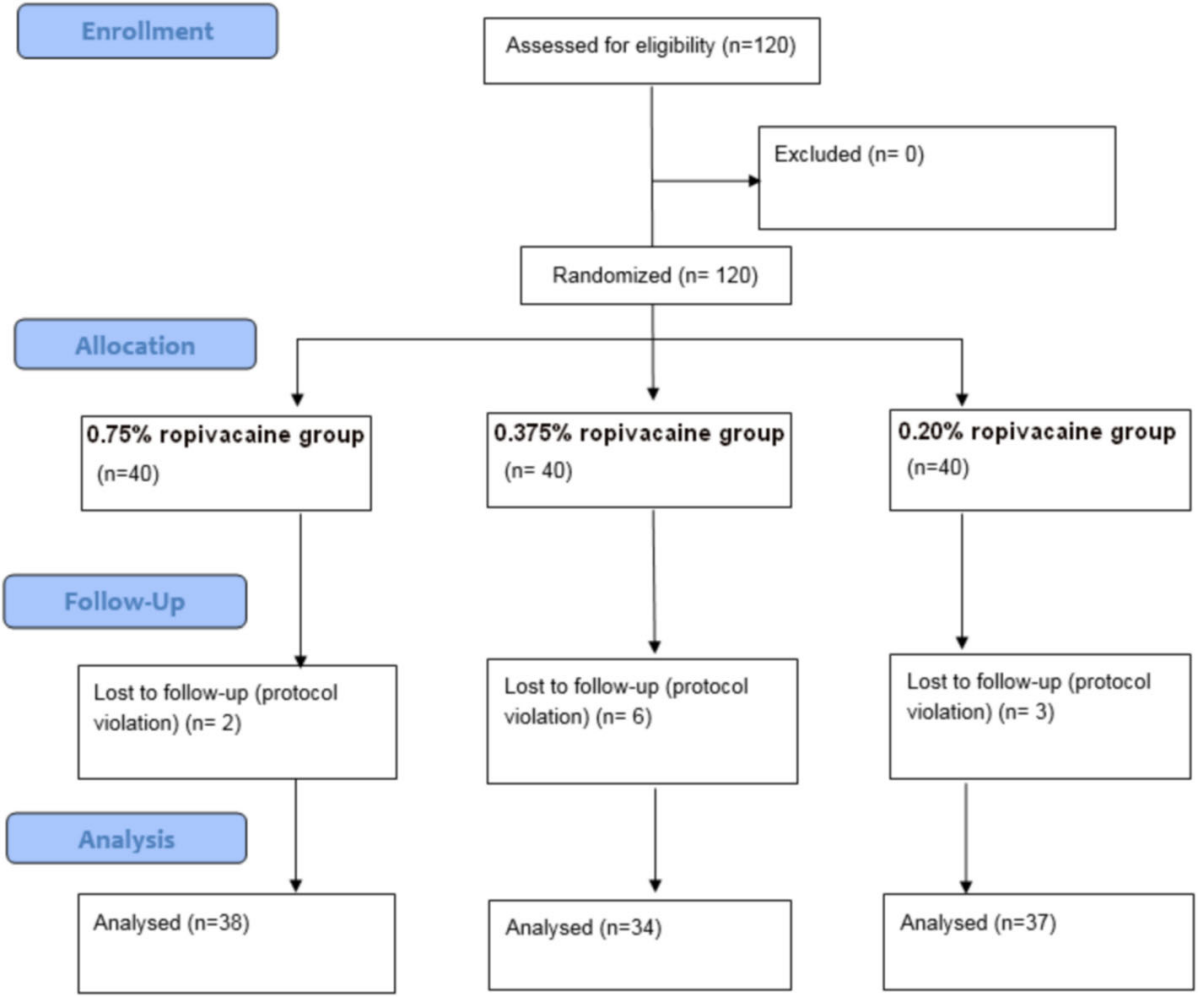
Consort flow diagram

The Consort flow diagram is shown in Fig. 1. A total of 109 patients were included for analysis. The demographic data are shown in Table 1 and there were no significant differences among the three groups. BIS and end-tidal sevoflurane concentration also showed no significant differences among the groups.

**Table 1:** Demographic Data

	0.75% Ropivacaine (*n* = 38)	0.375% Ropivacaine (*n* = 34)	0.20% Ropivacaine (*n* = 37)	*P* value
Age (yrs)	63.0 (12.0)	65.5 (17.0)	59.0 (19.3)	0.57
Height (cm)	164.9 (9.6)	162.8 (13.8)	161.0 (13.3)	0.37
Weight (kg)	62.7 (9.9)	62.4 (16.9)	60.6 (16.1)	0.74
Sex, n (%)				0.17
M	29 (76.3)	19 (55.9)	23 (62.2)	
F	9 (23.7)	15 (44.1)	14 (37.8)	
ASA PS, n (%)				0.24
I	6 (15.8)	9 (26.5)	12 (32.4)	
II	32 (84.2)	25 (73.5)	25 (67.6)	
Ephedrine, n (%)	16 (42.1)	6 (17.6)	8 (21.6)	0.04
Fasting time (min)	693.8 (178.6)	762.6 (121.2)	749.3 (126.9)	0.09
Mean fluid administration until the end of the study (ml)	972.6 (99.3)	987.9 (105.3)	956.1 (149.9)	0.54
Type of surgery, n (%)				0.82
Extended cholecystectomy	4 (10.5)	3 (8.8)	8 (21.6)	
Hepatectomy	17 (44.7)	15 (44.12)	15 (40.5)	
Gastrectomy	9 (23.7)	9 (26.5)	8 (21.6)	
Pylorus preserving pancreaticoduodecectomy	8 (21.1)	8 (20.6)	6 (16.2)	

In the 0.75% ropivacaine group, there was significantly decreased MAP, SVRI, and significantly increased SVV compared with those in the 0.375% and 0.2% groups.

Moreover, the proportion of patients who needed ephedrine was remarkably higher in the 0.75% ropivacaine group (Table 1).

**Fig. 2:**
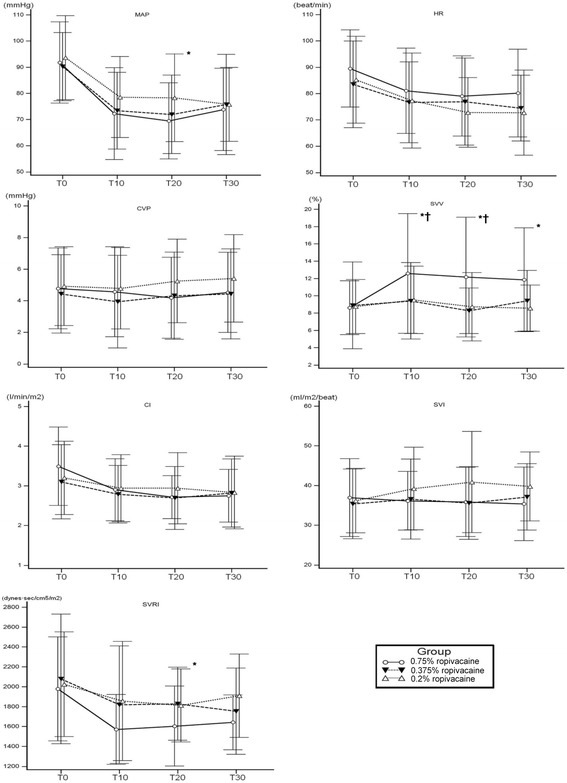
Hemodynamic changes following epidural administration of ropivacaine. MAP (*P* = 0.039) and SVRI (*P* = 0.026) were significantly decreased in 0.75% ropivacaine group compared with other groups. Concordant increase in SVV was remarkably increased in 0.75% group compared with 0.375% and 0.2% group through the study period (*P* = 0.017). * mean *P* < 0.05 compared with 0.2% group and † means P < 0.05 compared with 0.375% group

The time course of hemodynamic and parametric changes is shown in Fig. 2. Significant changes were observed at T10 (10 min after loading).

We also analyzed the differences in hemodynamic changes in young (<60 years) versus elderly (>60 years) patients in each group as shown in Table 2. In the 0.75% ropivacaine group, there were significant differences in CVP, SVV, SVRI, and SVI between the young and elderly subgroups.

**Table 2:** Subgroup analysis of hemodynamic data

	0.75% ropivacaine			0.375% ropivacaine			0.2% ropivacaine		
	< 60 yrs	> 60 yrs	*P* value	< 60 yrs	> 60 yrs	*P* value	< 60 yrs	> 60 yrs	*P* value
Age (yrs)	50.3 ± 10.5	68.0 ± 6.1		45.5 ± 14.8	69.1 ± 5.0		50.1 ± 9.7	70.3 ± 4.9	
MAP (mmHg)			0.190			0.577			0.851
Load	87.9 ± 13.2	94.1 ± 16.5		90.2 ± 14.8	90.1 ± 12.3		92.5 ± 17.9	94.7 ± 14.1	
10 min	76.1 ± 14.8	69.7 ± 18.9		71.2 ± 12.3	74.4 ± 15.9		75.8 ± 14.7	81.4 ± 16.0	
20 min	75.3 ± 16.7	65.7 ± 11.8		70.3 ± 15.5	72.7 ± 14.9		74.1 ± 15.3	82.7 ± 17.4	
30 min	76.0 ± 14.8	72.4 ± 16.3		81.0 ± 26.0	72.9 ± 14.3		73.8 ± 12.2	77.7 ± 15.9	
CVP (mmHg)			0.011			0.225			0.300
Load	4.7 ± 2.7	4.8 ± 2.4		4.1 ± 1.9	4.5 ± 2.7		5.8 ± 2.7	3.9 ± 1.8	
10 min	4.7 ± 2.7	4.4 ± 2.9		3.4 ± 2.3	4.2 ± 3.1		5.3 ± 2.7	4.1 ± 2.3	
20 min	4.5 ± 2.9	3.9 ± 2.3		3.6 ± 2.0	4.6 ± 3.0		5.3 ± 2.4	5.1 ± 2.8	
30 min	5.0 ± 2.8	4.1 ± 2.2		4.1 ± 2.5	4.5 ± 3.0		5.5 ± 2.9	5.2 ± 2.6	
SVV (%)			0.032			0.522			0.885
Load	7.5 ± 2.9	9.2 ± 3.0		10.6 ± 6.7	7.9 ± 3.6		8.2 ± 2.2	9.3 ± 3.7	
10 min	10.4 ± 5.8	13.8 ± 7.3		8.5 ± 2.0	9.8 ± 5.2		9.5 ± 3.6	9.5 ± 4.2	
20 min	8.9 ± 3.9	14.1 ± 7.6		7.7 ± 2.2	8.5 ± 2.8		7.6 ± 2.0	9.8 ± 5.0	
30 min	11.0 ± 6.2	12.3 ± 5.8		7.8 ± 2.7	10.2 ± 3.6		8.6 ± 2.3	8.5 ± 2.9	
SVRI (dynes-sec/cm^–5^/m^2^)			0.024			0.011			0.320
Load	1847.7 ± 500.1	2300.0 ± 634.6		2098.3 ± 660.7	2536.0 ± 659.3		2124.8 ± 558.0	2604.2 ± 634.1	
10 min	1811.4 ± 453.0	2026.5 ± 553.4		1898.6 ± 696.8	2189.9 ± 512.9		1828.0 ± 458.9	2456.8 ± 703.5	
20 min	1978.6 ± 374.4	1941.4 ± 344.0		1977.8 ± 747.1	2173.1 ± 282.9		1809.1 ± 407.1	2392.5 ± 469.1	
30 min	1967.1 ± 414.2	2140.2 ± 525.1		1945.0 ± 674.9	2185.6 ± 360.2		1888.4 ± 497.2	2342.4 ± 512.6	
SVI (mL/beat/m^2^)			0.011			0.002			0.342
Load	39.6 ± 9.0	35.2 ± 10.0		38.9 ± 11.4	33.5 ± 6.5		37.7 ± 8.7	34.4 ± 7.2	
10 min	38.4 ± 7.6	34.7 ± 6.9		39.3 ± 13.9	35.1 ± 7.2		41.7 ± 11.1	36.5 ± 9.0	
20 min	38.6 ± 8.9	34.1 ± 8.2		37.4 ± 11.2	34.6 ± 7.8		42.8 ± 11.7	38.8 ± 13.7	
30 min	37.7 ± 9.6	33.8 ± 8.8		40.8 ± 10.8	35.2 ± 6.0		41.8 ± 10.4	37.5 ± 5.9	
CI (L/min/m^2^)			0.901			0.082			0.512
Load	3.8 ± 0.9	3.2 ± 0.9		3.5 ± 1.1	2.8 ± 0.6		3.4 ± 0.8	2.9 ± 0.9	
10 min	3.3 ± 0.8	2.6 ± 0.6		3.1 ± 0.8	2.6 ± 0.5		3.2 ± 0.8	2.6 ± 0.6	
20 min	2.9 ± 0.6	2.5 ± 0.4		3.0 ± 1.0	2.5 ± 0.5		3.2 ± 1.0	2.6 ± 0.5	
30 min	2.9 ± 0.7	2.6 ± 0.5		3.3 ± 1.1	2.5 ± 0.4		3.1 ± 1.0	2.5 ± 0.6	
HR (beats/min)			0.025			0.776			0.119
Load	93.0 ± 13.0	87.3 ± 15.3		86.9 ± 12.6	81.6 ± 18.1		85.0 ± 13.2	85.5 ± 19.7	
10 min	83.5 ± 14.9	79.4 ± 17.0		74.0 ± 12.0	77.9 ± 16.9		76.9 ± 13.3	77.7 ± 22.4	
20 min	80.3 ± 18.1	78.2 ± 13.4		75.1 ± 16.1	77.8 ± 17.0		72.8 ± 11.5	72.7 ± 15.1	
30 min	80.1 ± 17.1	80.1 ± 16.6		77.5 ± 11.2	72.8 ± 13.0		74.5 ± 16.3	70.8 ± 16.2	
Total-ephedrine (mg)	10.0 ± 13.5	9.0 ± 14.4	0.833	0.4 ± 1.4	2.6 ± 6.1	0.238	0.5 ± 2.2	0.0 ± 0.0	0.337

The correlations between parameters are shown in Table 3. The MAP showed a negative correlation with SVV and a positive correlation with SVRI.

**Table 3:** Correlation analysis among haemodynamic parameters

		MAP	CVP	HR	CI	SVRI	SVI
CVP	Coefficient	0.074					
	*P*-Value	0.051					
HR	Coefficient	0.243	−0.208				
	P-Value	<0.0001	<0.0001				
CI	Coefficient	0.361	−0.133	0.469			
	P-Value	<0.0001	0.0004	<0.0001			
SVRI	Coefficient	0.273	−0.064	−0.186	−0.457		
	P-Value	<0.0001	0.093	<0.0001	<0.0001		
SVI	Coefficient	0.226	0.046	−0.351	0.584	−0.273	
	P-Value	<0.0001	0.223	<0.0001	<0.0001	<0.0001	
SVV	Coefficient	−0.244	−0.158	0.116	−0.214	0.087	−0.351
	P-Value	<0.0001	<0.0001	0.002	<0.0001	0.022	<0.0001

**Fig. 3:**
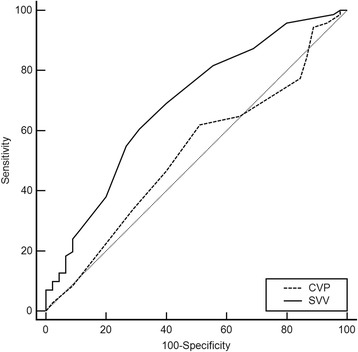
Receiver operating characteristics (ROC) curve analyses to predict change of mean arterial blood pressure after thoracic epidural anesthesia. The AUC of SVV was 0.687 (95% CI, 0.587–0.787) whereas AUC of CVP (0.477 [95% CI, 0.369–0.584]). The performance to predict 20% change of mean arterial pressure was significantly different (P = 0.026). The optimal cutoff value of SVV to discriminate between responders and non-responders was 9.5% (sensitivity: 60.6%, specificity: 68.9%)

Hemodynamic data comparing responders (20% decrease in MAP) and non-responders are shown in Table 4 and the ROC curve for performance of SVV and CVP in predicting hypotension is shown in Fig. 3. Although SVV increased in the responder group, SVV was not a significant predictor of hypotension.

**Table 4:** Hemodynamic data

Variable		Nonresponders (*N* = 43)		Responders (*N* = 66)		
		Mean	SD	Mean	SD	*P* value
Age (yrs)		59.3	15.2	61.7	11.4	0.341
BSA (m^2^)		1.6	0.2	1.7	0.2	0.150
MAP (mmHg)	T0	83.9	12.2	86.9	14.3	0.254
	T20	82.4	15.7	69.6	14.5	<0.001
HR (beats/min)	T0	88.1	18.1	85.3	14.4	0.355
	T20	81.8	17.9	76.4	15.4	0.087
CVP (mmHg)	T0	4.5	2.3	4.8	2.6	0.534
	T20	4.6	2.9	4.3	2.7	0.595
CI (L/min/m^2^)	T0	3.2	1.1	3.3	0.9	0.732
	T20	3.1	0.9	2.8	0.7	0.051
SVI (mL/beat/m^2^)	T0	36.5	8.3	36.1	9.4	0.806
	T20	38.3	9.8	36.6	8.9	0.318
SVRI (dynes-sec/cm^–5^/m^2^)	T0	2178.4	704.6	2359.9	597.4	0.215
	T20	2109.5	517.4	1895.7	411.4	0.015
SVV (%)	T0	8.2	3.2	9.1	4.1	0.258
	T20	8.7	4.0	11.80	6.0	0.003

## Discussion

In our study, we found the followings: 1) Significantly decreased MAP after epidural administration of ropivacaine was observed at a concentration of 0.75% ropivacaine, but not at concentrations of 0.375% and 0.2% ropivacaine, 2) A reduction in SVR and an increase in SVV showed a significant correlation with a decrease of MAP, but CVP did not, 3) In elderly patients, hypotension in the high concentration group was more prevalent and the accompanying SVV changes were more pronounced, 4) SVV was found to be a weak predictor of hypotension following TEA although it showed a significant correlation with MAP changes.

Hypotension is documented to be most common adverse effect after epidural administration of ropivacaine [10]. Hypotension occurs 5–20 min after the administration of epidural loading. The incidence of hypotension was significantly different depending on the concentration of ropivacaine; 54.6%, 49.2%, and 38.7% in 1%, 0.75%, and 0.5% of ropivacaine, respectively in the Food and drug administration (FDA) report. In our data, the incidence of hypotension was 61.2%, and a marked decrease in MAP was observed in 0.75% ropivacaine group compared to the other concentration groups. Some studies have reported conflicting results. Ginosar and colleagues did not find any differences between the groups administered 0.5% and 0.25% bupivacaine [6]. Dernedde and colleagues also observed that a 0.5% levobupivacaine group showed no statistically significant difference in hemodynamic parameters compared to 0.15% levobupivacaine, which was a much lower concentration than that used in our study [11]. Liu and colleagues also suggested that episodes and severity of hypotension and orthostatic changes in systolic blood pressure or heart rate were equivalent among the groups administered different concentrations of ropivacaine [12]. They investigated 0.05%–0.2% of ropivacaine with fentanyl during the study. We assumed that different concentrations and types of LA, site of blockade, and the combination with general anesthesia might cause these differences. Thus we should be aware of the possibility of hypotension in patients administered 0.75% ropivacaine epidurally during general anesthesia.

To date, hemodynamic changes after epidural anesthesia have been shown to be affected by various factors. The width of sympathetic denervation, balance of sympathetic and parasympathetic activity, pharmacological effect of systemically absorbed LA, and distribution of circulatory blood volume following epidural anesthesia should be taken into account when considering the hemodynamic effects of epidural anesthesia [6, 13–15]. Low thoracic epidural anesthesia (T5-L4), similar to our study, induces hypotension mainly by peripheral sympathetic blockade with block of the splanchnic fibers, whereas high thoracic epidural anesthesia (T1 to T5) induces hypotension by block of the cardiac afferent and efferent sympathetic fibers with loss of chronotropic and inotropic drive to the myocardium.

A reduction of MAP was regarded owing to a decrease in SVR. However, few clinical studies have investigated the change in SVR under TEA. Since the sympathetic blockade occurring after epidural anesthesia causes compensatory vasoconstriction of capacitance vessels of mesentery and lower extremity, it is difficult to study the change of SVR following TEA. In our study, we demonstrated a significant decrease of SVRI and accompanied decrease of MAP following epidural administration of 0.75% ropivacaine. But, we could not observe the difference in SVI and HR among the groups. Therefore more decrease of MAP in 0.75% ropivacaine was supposed to be a result of a more decrease in SVR by deeper sympathetic blockade, but not negative inotropic and chronotropic effect.

SVV, the variation in left ventricular stroke volume between the inspiratory and the expiratory phase during positive-pressure ventilation, is considered to be a good indicator of fluid responsiveness in the intensive care unit (ICU) and the operating room [16]. In our result, the elevation of SVV was accompanied by the decrease of SVRI, which was remarkable in 0.75% ropivacaine group. On the correlation analysis, SVV correlated negatively with MAP and positively with SVRI, not with CVP. Therefore, we can assume that increased SVV are the result of decreased SVRI by sympathetic blockade. In this case, increased SVV means functional hypovolemia and use of vasopressor could be preferable, not the volume expansion for the correction of accompanying hypotension.

The subgroup analysis demonstrated that hypotension in the high concentration group was more prevalent in elderly patients, and the accompanying SVV changes were more prominent. Therefore we can assume that a high dose of ropivacaine (0.75%) in elderly patients caused the increase of the block width and thus the hemodynamic effect of TEA was exaggerated. Previous many studies concerning the effect of age on spread after TEA documented segmental dose reduction is required with increasing age after TEA [17–20]. In addition, Stephen et al. described decrease in MAP after epidural lidocaine administration was significantly more in elderly patients [19]. The mechanism of increased block width in older patients is presumed to be a decreased egress of injected fluids via neural foramina in aged patients and increased susceptibility due to decrease in the number of myelinated nerve fibers and general deterioration of the mucopolysaccharides. It is assumed that increased susceptibility is also associated with prominent response at high concentrations in our study. In addition, SVI and HR in 0.75% group was significantly lower in elderly patients. We assumed that extended blockade to high thoracic level might cause negative inotropic and chronotropic effect in elderly patients. Another suspected mechanism is due to non-compliant heart of elderly patient [21]. Even small change in venous return will cause large change in ventricular preload and SV in non-compliant heart. Therefore we suggest that high concentration of ropivacaine should be avoided in elderly patients during TEA.

A few studies have reported SVV is a useful predictor of potential hypotension during the early postoperative period following a combination of general and epidural anesthesia. However, the reliability of SVV could still be an issue and needs to be further investigated during TEA. Kobayashi and colleagues suggested that SVV could predict fluid responsiveness in patients undergoing surgery with OLV (AUC, 0.900; optimal threshold value, 10.5%) [22]. Xu and colleagues also suggested that SVV could be an accurate indicator (AUC, 0.86; optimal threshold value, 13%) [23]. In our data, SVV was acceptable as an ancillary prediction tool (AUC, 0.687) and the optimal threshold to differentiate between responders and non-responders was 9.5% (sensitivity of 60.6% and specificity of 68.9%). However, according to a reported guideline for accuracy of a diagnostic system, the AUC should be above 0.7 [24]. Thus it could be considered weak as an indicator to predict subsequent hypotension in our study.

Our study has several limitations. First, the use of ephedrine during the study period for protection against possible adverse events following hypotension might mask the hemodynamic effect caused by epidurally administered ropivacaine. We analyzed the consumption and incidence of ephedrine used to correct for bias. The consumption of ephedrine was highest in the 0.75% ropivacaine group. Therefore, we thought a decrease of MAP and SVR might be more significant in the 0.75% ropivacaine group if ephedrine was not used. Although SVV could be affected by the use of vasoactive drugs such as ephedrine, Hadian reported that increasing inotropes or vasoconstrictors did not change SVV [25]. Therefore, the effect of ephedrine on SVV could be limited. Second, we did not assess the height of the epidural blockade because the study solution was administered during general anesthesia. Third, we did not show the hemodynamics at a fixed dose at a different concentration to determine the concentration effect on hemodynamics following TEA. We could not perform the study at a fixed dose because a high volume was needed to meet the 0.75% ropivacaine (60 mg) dose at a low concentration. We considered that the administered volume of LA could be an influencing factor on the block height and following hemodynamics in TEA. Additionally, other factors that could affect SVV such as intra-abdominal pressure, basal intra-vascular volume status, and intrinsic chance of error of SVV were not considered.

## Conclusions

In this study, we found that a concentration of 0.75% but not that below 0.375% of ropivacaine, administered epidurally with general anesthesia may be associated with significant hypotension, especially in patients older than 60 years. Therefore, care should be taken to prevent significant hypotension when epidural analgesia is performed with 0.75% ropivacaine under general anesthesia in elderly patients. An increase in SVV, but not changes in CVP, is significantly correlated with the development of hypotension. Although SVV was not found to be a strong predictor of hypotension after TEA, we still recommend a cutoff value of 9.5%.

## Abbreviations

AUC: Areas under curve
BIS: Bispectral index scale
BSA: Body surface area
CI: Cardiac index
CVP: Central venous pressure
FDA: Food and drug administration
HR: Heart rate
ICU: Intensive care unit
LA: Local anesthetics
MAP: Mean arterial blood pressure
ROC: Receiver operating characteristic
SVI: Stroke volume index
SVRI: Systemic vascular resistance index
SVV: Stroke volume variation
TEA: Thoracic epidural anesthesia
